# A Multi-Modal Approach to Closing Exploratory Laparotomies Including High-Risk Wounds

**DOI:** 10.7759/cureus.9087

**Published:** 2020-07-09

**Authors:** Erin G Andrade, Jarot J Guerra, Laurie Punch

**Affiliations:** 1 Surgery, Washington University, St. Louis, USA; 2 Surgery, Rutgers New Jersey Medical School, Newark, USA; 3 Surgery, Barnes-Jewish Hospital, Washington University, St. Louis, USA

**Keywords:** surgical site infection, laparotomy, closed incision negative pressure wound therapy, emergent general surgery

## Abstract

Background

Laparotomy incisions with contamination have a high incidence of surgical site infection (SSI). One strategy to reduce SSI has been to allow these wounds to heal by secondary intention; however, this results in an ongoing need for wound care after discharge.

Methods

A prospectively maintained Acute and Critical Care Surgery database was queried for patients who underwent exploratory laparotomy during 2008-2018. Patients were stratified into two groups: 2008-2015 (no protocol [NP]) and 2016-2018 (closure protocol [CP]). CP patients were operated on by a single surgeon utilizing a multi-modal high-risk incisional closure protocol, which included dilute chlorhexidine lavage, closed suction drains for incisions deeper than 3 centimeters, and incisional negative-pressure wound therapy (iNPWT). The CDC (Centers for Disease Control and Prevention) guidelines were used to determine wound classification and SSI based on chart review. Groups were compared using univariate and multivariate analysis.

Results

A total of 139 patients met the study criteria. The overall SSI rate, including superficial and deep space infections, was no different in NP versus CP (21.6 vs. 24.1%; p=0.74). The rate of superficial SSI was similar between NP and CP (11.8 vs. 8.4%; p=0.53). Rates of wound closure at discharge were higher in the CP group than the NP group across wound classes, with the greatest difference among dirty wounds (50.0% NP vs. 94.9% CP; p<0.01). CP significantly increased the likelihood of wound closure (OR=179.2; p<0.001) even after controlling for body mass index, wound classification, ASA (American Society of Anesthesiologists) status, and initially open abdomen.

Conclusions

By addressing both tissue factors and bacterial burden through the use of a multi-modal high-risk incisional closure protocol involving iNPWT, all wounds can be considered for closure without increasing the risk of SSI.

## Introduction

Surgical site infections (SSIs) represent both the most prevalent and most costly form of nosocomial infection in the United States, occurring after 2-5% of all operations and more than 16% of abdominal operations annually [1,2]. Public reporting of SSI incidence rates is a required quality indicator used by the Centers for Medicare and Medicaid Services [3]. It has been estimated that 55% of SSI are preventable through the use of best practices [4]. Efforts to reduce SSI are common in the surgical literature but vary based on wound classification.

Healing by secondary intention is often used in high-risk wounds to decrease rates of SSI [5]. Wounds may be high-risk for infection secondary to patient factors (age, obesity, smoking, malnutrition, diabetes mellitus), tissue characteristics (ischemia, dead space), and level of bacterial contamination. Although healing by secondary intention reduces SSI, it increases healthcare costs [6,7]. Historically, delayed primary closure has been used to mitigate the risk of SSI in contaminated or dirty wounds by avoiding closure at the initial operation when the bacterial burden is higher, while still eventually achieving a primarily closed wound [8,9]. However, not all studies show a decrease in SSI with delayed primary closure [10,11].

While many have addressed patient factors pre-operatively to reduce SSI rates, this is rarely possible in emergent general surgery cases [12,13]. However, tissue characteristics and level of contamination can be addressed through a multi-modal approach. Prior studies have shown that subcutaneous wound depth and obesity are risk factors for SSI [14-16]. Obesity increases the risk of SSI even in clean and clean-contaminated cases, where SSIs are generally rare [16]. Because patient risk factors, such as wound depth and obesity, may make a wound high-risk for complications beyond the risk typically associated with a given wound classification, we developed a multi-modal protocol that identifies wounds at high-risk for wound complications including SSI and dehiscence across all wound classifications based on the following high-risk criteria: body mass index (BMI) greater than 30, delayed primary closure, creation or revision of an ostomy, or the presence of an enterocutaneous fistula. Our closure protocol seeks to counteract high-risk tissue characteristics through incisional negative-pressure wound therapy (iNPWT), selective use of closed suction drains in deep wounds, and reduction of contamination level through dilute chlorhexidine lavage. The use of iNPWT increases oxygenation through angiogenesis, reduces tension on wound edges, and lowers the risk of seroma formation [17]. In deep wounds, in addition to iNPWT, closed suction drains were added to further decrease seroma risk. Finally, chlorhexidine gluconate, both an effective biocide and a surfactant, was lavaged to decrease the wound’s bioburden [18,19].

We hypothesized that the use of the above multi-modal high-risk incisional closure protocol could increase rates of primary closure without increasing rates of SSI.

## Materials and methods

**Figure 1:**
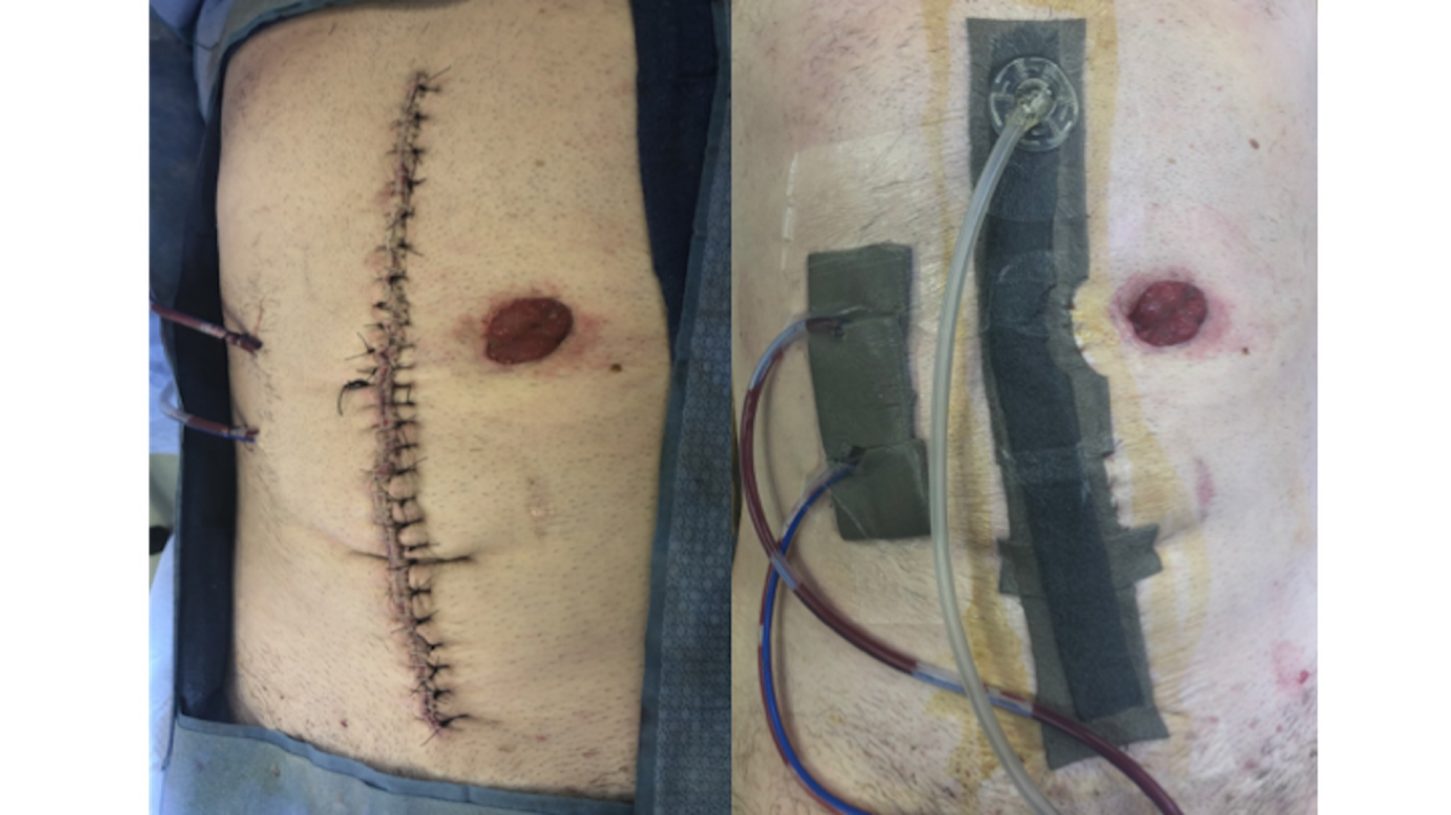
Incisional Negative-Pressure Wound Therapy with PolyMem Wic Silver®

A prospectively maintained acute care surgery database spanning 2008-2018 was queried for patients who underwent exploratory laparotomy using ICD-9 (International Classification of Diseases, Ninth Revision) codes. Trauma patients and in-hospital deaths were excluded. Patients were stratified into two time periods: 2008-2015 (no protocol [NP]) and 2016-2018 (closure protocol [CP]). In the NP group, all exploratory laparotomies performed by surgeons in the acute care division were included. Patients were closed according to usual practice with staples or subcuticular absorbable suture according to surgeon preference. Contaminated wounds were lavaged with saline prior to closure. Within the NP group, no iNPWT was used. In the CP period, patients were operated on by a single surgeon, who was piloting the use of a multi-modal incisional closure protocol. Within the CP group, patients were deemed high-risk if they met any of the following criteria: BMI greater than 30, delayed primary closure, creation or revision of an ostomy, or the presence of an enterocutaneous fistula. After fascial closure, high-risk patients underwent dilute chlorhexidine lavage of the soft tissue, placement of subcutaneous closed suction drains for incisions deeper than 3 centimeters, and iNPWT (Figure 1). Incisional NPWT was created using PolyMem WIC Silver as a base over the closed incision, with black foam placed on top.

Study variables including patient demographics, medical co-morbidities, hospital length of stay, re-admissions, wound class, and the presence of SSI were manually extracted from the electronic medical record. The wound classification was determined by review of the operative report and application of the Centers for Disease Control and Prevention (CDC) definition [20]. SSIs were defined as a wound infection occurring within 30 days of surgery and subdivided into superficial incisional, deep incisional, and organ space infections based on the CDC guidelines; for open wounds, superficial SSIs were defined as wounds having erythema or purulence that were determined to require antibiotics by the treating physician [20]. Complications were defined as any SSI, return to emergency room, re-admission, or wound dehiscence within 30 days.

Statistical analysis was performed using Stata 14.3 (StataCorp LLC, College Station, TX, USA). Categorical variables were analyzed using Pearson’s chi-square test to determine statistical significance. Student’s t-test was used to determine statistical significance for continuous variables, which are reported as mean and standard deviation. Multivariate logistic regression models were used to assess the impact of CP on skin closure considering known risk factors for SSI as possible confounders. A step-wise approach was used with any variable, with p<0.20 remaining in the model. P-values less than 0.05 were considered significant.

## Results

During the study period, 170 patients underwent exploratory laparotomy for a non-traumatic indication. After 31 patients were excluded for in-hospital mortality, 52 patients remained in the NP group and 87 in the CP group. Demographics between the two groups were similar (Table 1). The CP group contained higher risk patients both in terms of higher mean BMI (26.6 vs. 30.6; p=0.01) and higher proportion of patients with the American Society of Anesthesiologists (ASA) class III or higher (Table 1). Additionally, the CP group contained a greater proportion of dirty cases compared with the NP group (44.8% vs. 26.9%; p=0.002; Table 2). Within the NP group, 42.0% met at least one of the criteria for high-risk closure according to the high-risk incisional closure protocol compared with 67.8% if the CP group (p<0.01). Compliance with the high-risk protocol among the CP patients who met high-risk criteria was 84.8% (50/59); an additional 13 patients who did not meet high-risk criteria also underwent high-risk protocol.

**Table 1:** Demographic Characteristics and Co-morbidities SD, standard deviation; ASA, American Society of Anesthesiologists

Variable	No Protocol, n = 52	Closure Protocol, n = 87	p-Value
Age, years (SD)	57.7 (15.4)	58.1 (16.4)	0.89
Male (n)	44.2% (23)	44.8% (39)	0.95
Smoker (n)	26.5% (13)	22.6% (19)	0.61
Diabetes mellitus (n)	17.3% (9)	28.7% (25)	0.13
Steroid use (n)	10.2% (5)	13.8% (12)	0.54
Body mass index (SD)	26.6 (7.1)	30.6 (10.0)	0.01
ASA class III or greater (n)	66.0% (33)	85.1% (74)	0.01

**Table 2:** Case Volume by Wound Classification

	No Protocol	Closure Protocol	p-Value
Clean	40.4% (21)	12.6% (11)	0.002
Clean-contaminated	21.2% (11)	25.3% (22)	
Contaminated	11.5% (6)	17.2% (15)	
Dirty	26.9% (14)	44.8% (39)	

**Figure 2:**
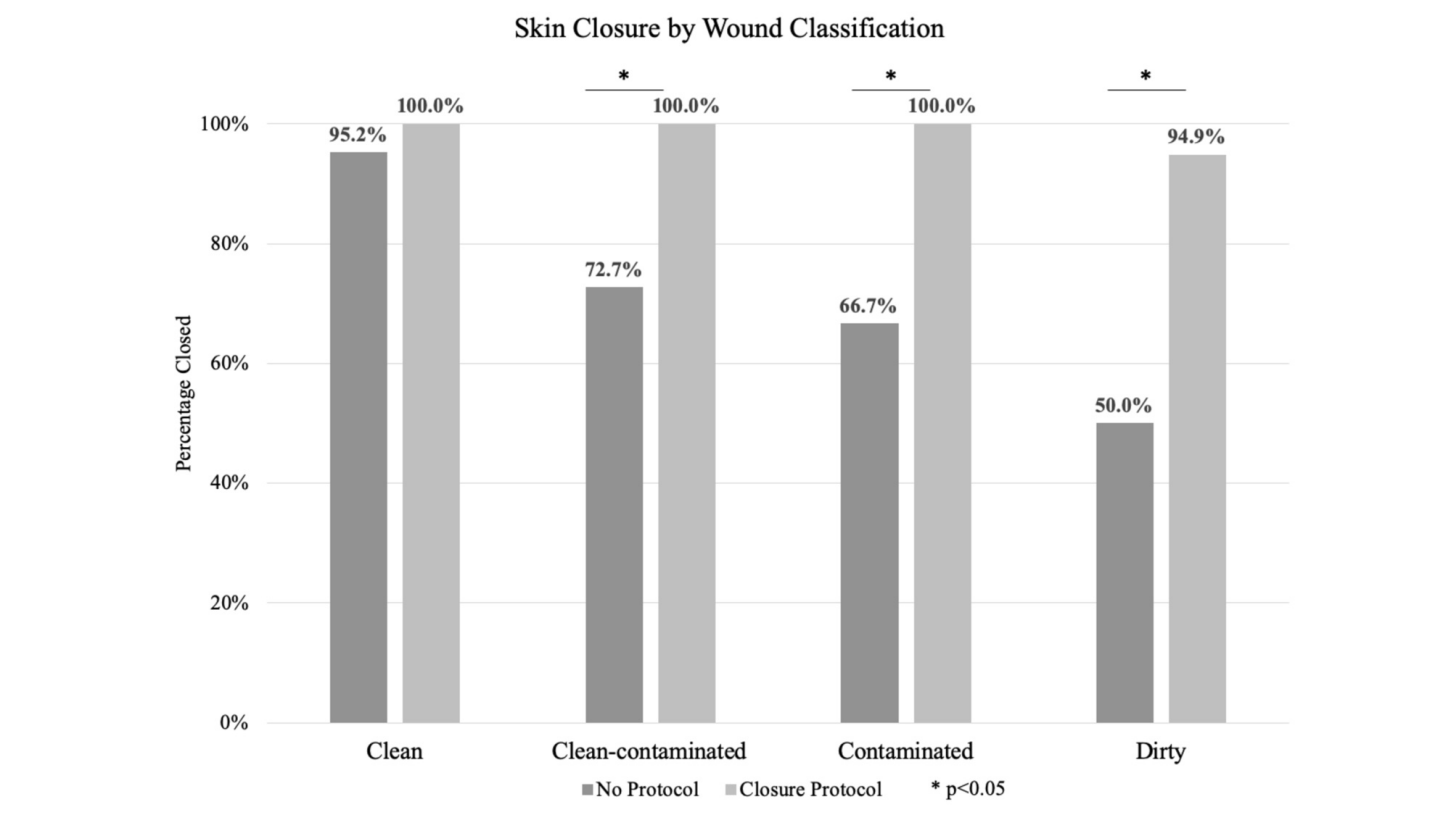
Skin Closure Rate by Wound Classification with and without Closure Protocol

Both groups had similar proportions of patients with an open abdomen after their initial operation (25.0% NP vs. 27.6% CP; p=0.74; Table 3). Reasons for not closing the fascia at the time of initial operation included hemodynamic instability, marginally ischemic bowel requiring a second look operation, and bowel edema preventing closure. In the NP group, 13.5% of patients underwent delayed primary closure versus 26.4% in the CP group (p=0.07). At the time of final operation, 97.7% in the CP group had their skin closed compared with 75.0% in the NP group (p<0.01; Figure 2). Despite having higher risk patients in the CP group, the overall SSI rate was not significantly different in NP versus CP (21.6% vs. 24.1%; p=0.74). This was also true for superficial SSI (11.8% NP vs. 8.4% CP; p=0.53). Thirty-day complication rates were not significantly different between the groups (46.2% NP vs. 44.1% CP; p=0.81). While the NP had a higher rate of emergency room visits within 30 days than the CP group (28.9% vs. 14.9%; p=0.05), both groups had relatively high readmission rates, with 28.9% in the NP group and 21.8% in the CP group getting readmitted within 30 days (p=0.47). Stratified by wound class, the CP group had higher rates of wound closure in every wound class (Figure 1). The greatest difference in closure rates was seen in dirty cases. Despite closing high-risk wounds, the CP group had similar rates of superficial skin dehiscence to the NP group (7.6% vs. 5.1%; p=0.62).

**Table 3:** Clinical Management and Outcomes SD, standard deviation; SSI, surgical site infection; ER, emergency room

Variable	No Protocol	Closure Protocol	p-value
Initially open (n)	25.0% (13)	27.6% (24)	0.74
Length of stay, days (SD)	17.4 (29.5)	20.9 (16.6)	0.38
Any SSI (n)	21.6% (11)	24.1% (20)	0.74
Superficial SSI (n)	11.8% (6)	8.4% (7)	0.53
Skin closed (n)	75.0% (39)	97.7% (85)	<0.01
30-day return ER (n)	28.9% (15)	14.9% (13)	0.05
30 day readmission (n)	28.9% (15)	21.8% (19)	0.35
30-day complications (n)	46.2% (24)	44.1% (37)	0.81

A step-wise multivariate logistic regression evaluated the association of CP on skin closure with the inclusion of wound classification, BMI, ASA class, smoking, and initially open abdomen as possible confounders. CP significantly increased the likelihood of wound closure at discharge (OR=179.2; p<0.001) (Table 4). Wound classification of contaminated or dirty and having an initially open abdomen were both negatively associated with skin closure.

**Table 4:** Logistic Regression Skin Closure OR, odds ratio

Variable	OR	95% Confidence Interval	p-value
Closure protocol	179.23	10.02-3206	<0.001
Wound class ≥ III	0.060	0.006-0.64	0.02
Initially open	0.028	0.027-0.28	0.002

## Discussion

These results suggest that the use of a multi-modal incisional closure protocol in high-risk exploratory laparotomy patients utilizing iNPWT allows for the closure of nearly all wounds. Despite closing wounds that are high-risk for SSI, there was no difference in overall or superficial SSI rates in the CP group compared with the NP group. Furthermore, the rate of wound dehiscence was equivalent.

Retrospective studies have shown the benefit of iNPWT in reducing rates of SSI after laparotomy for gynecologic and general surgery patients [21,22]. However, the results of randomized controlled trials have been mixed. A randomized trial of iNPWT in obese patients undergoing cesarean reported a reduction in SSI rates [23]. Another randomized trial of elective and urgent general surgery patients showed lower rates of SSI with iNPWT, but patients with dirty wounds, BMI > 40, and ASA > III were excluded [24]. Randomized controlled trials of iNPWT in laparotomy for colorectal surgery or for intra-abdominal malignancy both showed no difference in the SSI rate [25,26]. Our study, similar to these randomized controlled trials that included high-risk patients undergoing laparotomy, showed no difference in the SSI rate with the use of iNPWT.

Although the alternative to iNPWT presented in most studies is a dry dressing over a closed incision, in emergent general surgery cases often the alternative is leaving the wound open to heal by secondary intention or performing delayed primary closure. A small randomized trial of iNPWT versus open NPWT after laparotomy in contaminated and dirty wounds showed no significant difference in SSI and more rapid healing in the iNPWT group [27]. A prospective study of iNPWT in high-risk emergent general surgery patients showed a superficial SSI rate of 7.4% [28], similar to the 8.4% rate seen in our study. In addition to SSI rates, it is important to consider wound closure as an outcome due to the increased cost of persistently open wounds. Acker et al. showed decreased SSI in trauma laparotomies that were allowed to heal by secondary intention, as well as higher costs [7]. In addition to direct costs, persistently open wounds may lead to indirect costs to patients and their families due to loss of independence, decreased mobility, and depression [29].

This study has multiple limitations. The data are retrospective and limited by what is documented in the chart. The external validity of the results is limited as the CP patients were operated on by a single surgeon at a single institution as part of a pilot study. Moreover, the use of a historical control group introduces the potential for confounding as other unknown factors in management may have changed, which could have affected the outcome of wound closure. However, a historical rather than concurrent control group was chosen because during the closure protocol time period, other surgeons in the department were adopting some aspects of the protocol but not others, which would have limited the ability to draw conclusions about the effects of the multi-modal protocol. As this study involves a multi-modal protocol, it is impossible to determine which aspects of the protocol lead to increased probability of closure. In the future, a multi-pronged randomized controlled trial will be required to determine which aspects of the closure protocol are necessary to prevent increased rates of SSI in high-risk groups.

## Conclusions

By addressing both tissue factors and bacterial burden through the use of a multi-modal high-risk incisional closure protocol, all wounds can be considered for closure without increasing the risk of SSI or dehiscence. Given the limitations of this single-surgeon experience, further prospective study is needed.
